# Strength Profile Pattern of FRP-Reinforced Concrete Structures: A Performance Analysis through Finite Element Analysis and Empirical Modeling Technique

**DOI:** 10.3390/polym13081265

**Published:** 2021-04-13

**Authors:** Ali Raza, Syyed Adnan Raheel Shah, Hatem Alhazmi, Muhammad Abrar, Samia Razzaq

**Affiliations:** 1 Department of Civil Engineering, University of Engineering and Technology, Taxila 47080, Pakistan; araza4846@gmail.com; 2 Department of Civil Engineering, Pakistan Institute of Engineering and Technology, Multan 66000, Pakistan; 3 National Center for Environmental Technology (NCET), Life Science and Environment Research Institute (LSERI), King Abdulaziz City for Science and Technology (KACST), Riyadh 11442, Saudi Arabia; halhazmi@kacst.edu.sa; 4 Department of Electrical Engineering, Bahauddin Zakariya University, Multan 66000, Pakistan; mabrarbari@gmail.com; 5 School of Aerospace, Mechanical and Mechatronic Engineering, The University of Sydney, Camperdown 2006, Australia; Sraz2603@uni.sydney.edu.au

**Keywords:** glass fiber reinforced polymer, axial capacity, finite element analysis, concrete columns, parametric study, coefficient of determination

## Abstract

Limited research work is available in the literature for the theoretical estimates of axial compressive strength of columns reinforced with fiber reinforced polymer (FRP) rebars. In the present work, an experimental database of 278 FRP-reinforced concrete (RC) compression members was established from the literature to recommend an empirical model that can accurately predict the axial strength (AS) of GFRP-RC specimens. An initial assessment of 13 different previously anticipated empirical models was executed to achieve a general form of the AS model. Finally, a new empirical equation for forecasting the AS of GFRP-RC short columns was proposed using the curve fitting and regression analysis technique. The performance of the proposed empirical model over the previous experimental database represented its higher accuracy as related to that of other models. For the further justification of the anticipated model, a numerical model of GFRP-RC columns was simulated using ABAQUS and a wide parametric study of 600 GFRP-RC samples was executed to generate a numerical database and investigate the influence of various parameters using numerical and empirical models. The comparison between theoretical and numerical predictions with R^2^ = 0.77 indicted that the anticipated empirical model is accurate enough to apprehend the AS of FRP-RC specimens.

## 1. Introduction

The high maintenance costs and limited-service life of conventional steel reinforcement in aggressive and corrosive environments have spurred the interest of modern research in advanced composite materials such as fiber-reinforced polymers (FRPs). The higher tensile strength, lower density, lighter weight, lower maintenance costs, higher resistance to corrosion, lower conductance to temperatures, and high resistance to chemical environments are the main advantages of FRPs [1,2,3,4,5]. These days, the construction industry is focusing on the replacement of corrosive steel reinforcement with the FRP rebars and confinements due to their superiorities over steel rebars to minimize the effects of corrosion in aggressive and corrosive environments [6,7,8,9]. Most of the concrete bridges in the United States and Canada employed the FRP reinforcement as a partial or total replacement of steel rebars [10]. Although, the use of FRPs in the construction industry has been increased still no design guidelines have been added in North American codes for such reinforcement. Furthermore, the use of FRP rebars as longitudinal compressive reinforcements has been prevented in Canadian codes [11,12] due to the limited research and advancement in this area. This may also be associated with the uncertain performance of FRP rebars in compression and limited experimental data.

During the last few decades, for providing the flexural and shear reinforcements, the employment of FRP rebars in reinforced concrete members is increasing [13,14]. Some investigations have been carried out to examine the performance of FRP rebars as longitudinal and transverse reinforcements in compressive and flexural members which depicted a better response of FRP rebars in these members [15,16,17,18,19,20,21]. As the axial compressive performance of FRP rebars is lower than that of steel rebars, therefore, some investigations have been carried out to determine the strength reduction coefficients for FRP rebars to secure the most optimum results under compressive loads [22,23,24,25]. But there is still a need for refining these coefficients using an experimental database consisting of various sample points. The previously suggested models for the axial strength of columns reinforced with FRP rebars have deficiencies such as they were suggested based on small data points, the axial contribution of FRP rebars was not included in the axial compressive performance and bending performance and the compressive and tensile properties of FRP rebars were assumed to be the same. The axial strength (AS) is significantly improved by improving the lateral confinement of the concrete core but the steel reinforcement performs better than FRP reinforcement in compressive members after increasing the lateral confinement of the core [26,27,28]. The predictions are underestimated by neglecting the axial influence of FRP bars in columns while the predictions portray a close agreement with the experimental outcomes by considering the influences of AS and axial stiffness of FRP rebars [17,29,30,31].

Mohamed et al. [32] examined the behavior of fourteen (14) sand-coated FRP-reinforced columns confined with FRP ties/spirals under axial compressive load. Using smaller ratios of transverse reinforcement (0.7%), damage of the specimens happened due to buckling of longitudinal rebars. Similarly, using moderate ratios of transverse FRP confinement (1.5% and 2.7%), the damage of specimens happened due to the damage of spirals and the crushing of the core. Afifi et al. [33] anticipated an equation for GFRP-confined concrete based on the criterion of Willam-Warnke. This model was adjusted utilizing the regression analysis method on the experimental testing outcomes for envisaging the ultimate AS and corresponding axial strain of GFRP-RC columns. Twelve (12) circular GFRP-RC columns (with 205 mm diameter and 800 mm height) were studied by Hadi et al. [34] under various loading circumstances. The testing results depicted that the bending moment capacity and AS of GFRP-RC compression members were less than their identical steel-RC columns. Additionally, ignoring the influence of GFRP bars in the loading strength of columns origins an extensive discrepancy between the testing measurements and analytical predictions. Karim et al. [35] proposed a model for forecasting the axial load-deflection performance of GFRP-RC columns confined with GFRP spirals. Moreover, the effect of spiral-pitch and external GFRP sheets was also investigated. There was observed a two-peak axial loading performance of GFRP-RC columns confined with sheets; the first peak represented the axial capacity of concrete cross-section and the second represented the axial capacity of FRP-confined concrete core.

As concerned with the finite element analysis (FEA) of FRP-reinforced compressive members, a large of studies could be found in the literature that investigates the structural performance of such members using FEA under various conditions [5,19,20,30,36,37,38,39,40,41]. From these studies, it was detected that the projected FEA models captured the structural behavior of FRP-reinforced concrete compressive members precisely. The finite element models (FEM) represent all the shortages of the empirical models. In comparison with the experimental work, finite element simulation saves time and cost by generating computational models that can correctly capture the complex damage behavior of composites [42]. As FEA consumes huge time during the simulations that can be minimized by making some assumptions but these assumptions should be in such a way that they should not affect the accuracy of the models and give close estimations with the experimental outcomes at the same time. One should keep a balance between the analysis time, complexity of the models, size of the elements, and different types of elements in the models. Thus, FEA having a strong background knowledge is a very important and effective tool for the analysis of the structural engineering problems related to composites [43].

### Scope and Significance

It was observed from the literature review that there is a lack of studies on predicting the axial compressive strength of FRP-reinforced columns confined with FRP composites. Due to insufficient research data in this area, various international guidelines do not endorse the usage of such reinforcement in concrete columns. Therefore, detailed research is required in this area to provide some design guidelines for the structural performance of FRP-reinforced members under compressive loads. The main aims of the present investigation are: (a) To suggest a novel theoretical model for apprehending the axial compressive strength of FRP-reinforced concrete compressive elements based on various testing outcomes collected from the previous works; (b) to suggest a novel FEA model for accurately apprehending the compressive performance of such members with a minimum time; and (c) a detailed parametric investigation of GFRP-reinforced elements to observe the influence of various geometric and material variables of such members. To propose a novel theoretical model, an evaluation of the previous model over the constructed database has been performed to select the most suitable form. To perform FEM, a modified concrete damage plastic (CDP) model [30] is used for the simulations of the complex performance of concrete and the FRP bars are considered as a linear elastic material. The FEM has been proposed for the validation and comparison purposes of the newly anticipated theoretical model. The experimental results for the calibration and validation of FEA models have been taken from the literature [29]. Besides, widespread parametric training was accomplished using the anticipated FEM and empirical model to generate the results for the validation and comparison of the proposed models. A close agreement was observed between the predictions of the proposed empirical equation and FEM. The currently proposed empirical model has superiority over the previously suggested models for predicting the axial strength of GFRP-reinforced columns because it has been proposed over the large experimental dataset giving more accuracy and considering a large number of parameters of specimens. Furthermore, it is simpler and easier for practical applications. This research work is helpful for the analysis of GFRP-reinforced columns for the concrete construction industry.

## 2. Materials and Methods

### Database for Empirical Modeling

Many experimental research investigations have been done in the literature to study the axial performance of GFRP-RC columns. In the present research, a database of 278 FRP-RC columns was created from different research papers. The longitudinal FRP rebars and steel ties, steel spirals, FRP ties, or FRP spirals were employed as the longitudinal and transverse reinforcements in all the specimens in the constructed database. In the database, ten (10) columns were transversely unconfined, seven (7) columns were transversely reinforced with CFRP spirals, one hundred and ten (110) columns were transversely reinforced with GFRP spirals, hundred (100) columns were transversely reinforced with GFRP ties, eighteen (18) columns were transversely reinforced with steel spirals, and thirty-three (33) columns were transversely reinforced with steel ties. Various parameters have been included in the development of the database such as transverse reinforcement ratio ($\rho_{t}$), the elastic modulus of FRP bars ($E_{f}$), concrete compressive strength ($f_{c}^{\prime }$), longitudinal FRP reinforcement ratio ($\rho_{l}$), the ultimate tensile strain of FRP bars ($\varepsilon_{u}$), the tensile strength of FRP bars ($f_{u}$), breadth (B), width (H), and axial loading capacity ($P_{n}$) of specimens. Table 1 reports all the statistics of the parameters of the created database (provided in Appendix A).

## 3. Evaluation of Previous Models

**Figure 1:**
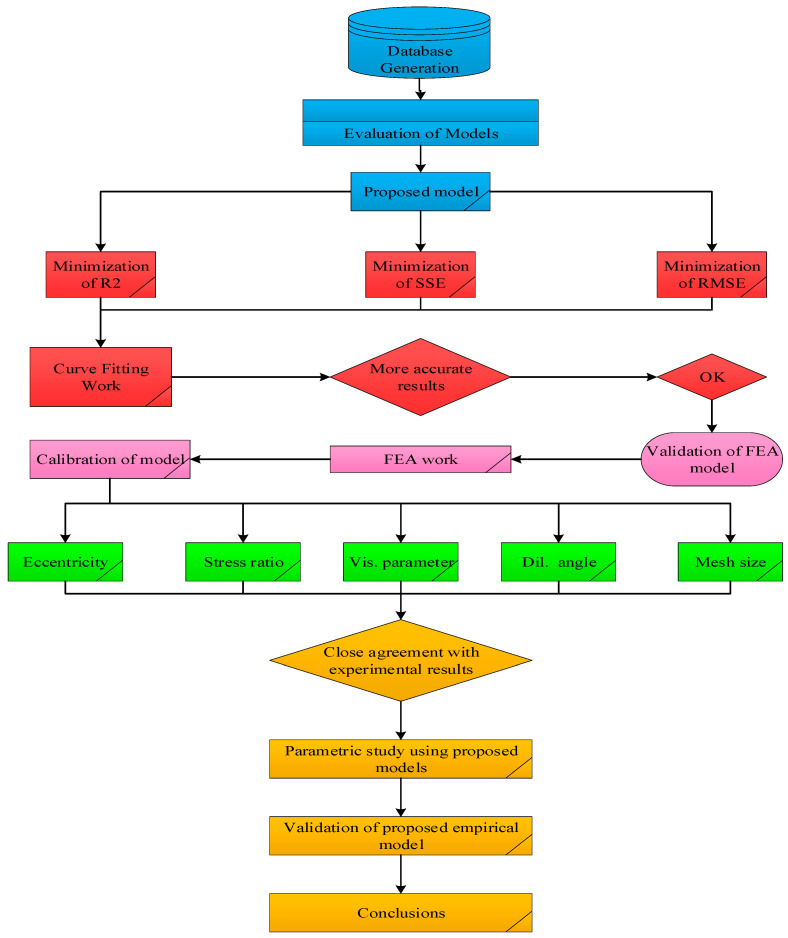
Flow chart of the present work.

Thirteen (13) existing models were assessed on the constructed database of GFRP-RC columns for selecting the most appropriate form of the newly proposed model. All the models that have been assessed for proposing the new general form of the model, are reported in Table 2. Three different statistical parameters (root mean squared error (RMSE), coefficient of determination (R^2^), and the mean absolute error (MAE)) were employed for the assessment of the models as reported by Equations (1)–(3). R^2^ is the most important parameter for examining the accuracy of a proposed theoretical model, therefore, the assessment of the models has been focused on using this parameter in the present research. The flow chart reported in Figure 1 presents the methodology of this investigation.

(1) $$R^{2}={\left(\frac{n\left(\sum_{i=1}^{n}x_{i}y_{i}\right)-\left(\sum_{i=1}^{n}x_{i}\right)\left(\sum_{i=1}^{n}y_{i}\right)}{\sqrt{\left[n\sum_{i=1}^{n}x_{i}{}^{2}-{\left(\sum_{i=1}^{n}x_{i}\right)}^{2}\right]\left[n\sum_{i=1}^{n}y_{i}{}^{2}-{\left(\sum_{i=1}^{n}y_{i}\right)}^{2}\right]}}\right)}^{2}$$

(2) $$MAE=\frac{1}{n}\overset{n}{\underset{i=1}{\sum }}\left|x_{i}-y_{i}\right|$$

(3) $$RMSE=\sqrt{\frac{1}{n}\overset{n}{\underset{i=1}{\sum }}{\left(x_{i}-y_{i}\right)}^{2}}$$

**Figure 2:**
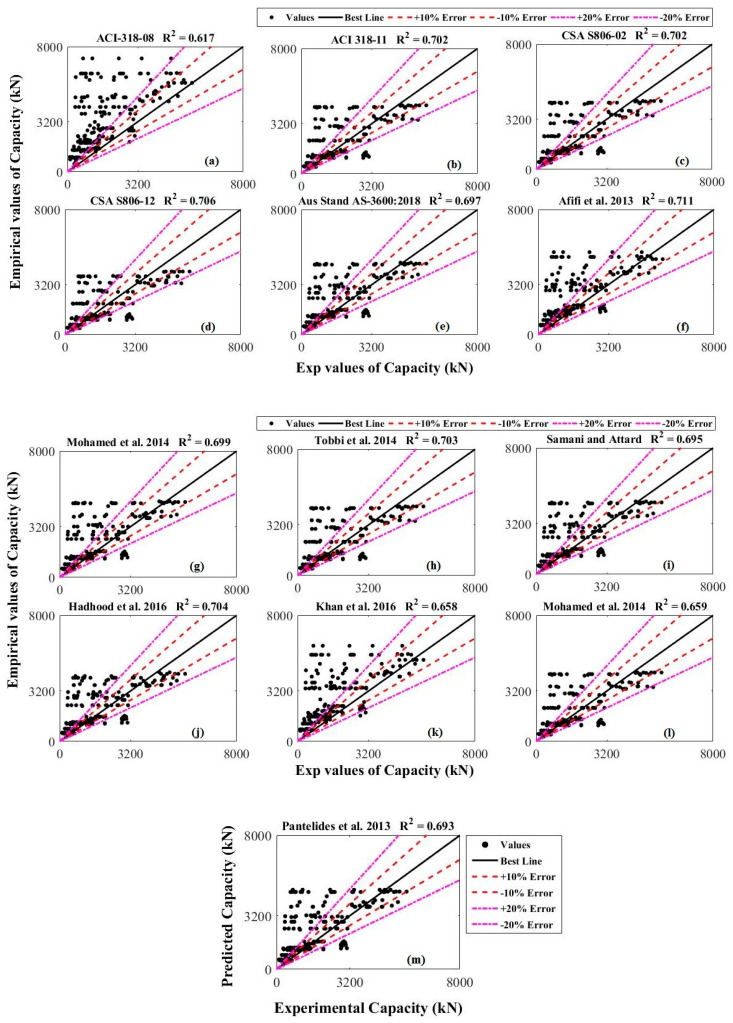
Performance of previous models (**a**) ACI 318-08 (**b**) ACI 318-11 (**c**) CSA S806-02 (**d**) CSA S806-12 (**e**) AS 3600-18 (**f**) Afifi et al. [23] (**g**) Mohamed et al. [32] (**h**) Tobbi et al. [48] (**i**) Samani and Attard [46] (**j**) Hadhood et al. [52] (**k**) Khan et al. [47] (**l**) Mohamed et al. [32] (**m**) Pantelides et al. [49].

In these expressions, n reports the number of test points, $x_{i}$ reports the AS taken from experiments and $y_{i}$ reports the AS taken from the empirical models. Figure 2 depicts the evaluations of the previous models over the database. These evaluations portray that the best accuracy is given by the Afifi et al. [23] model with the maximum value of R^2^ i.e., R^2^ = 0.711. If R^2^ is close to one (1.0), it will report a good correlation of theoretical estimates with the experimental outcomes. A value close to zero will report a week performance of the theoretical model. Due to the highest performance of the Afifi et al. [23] model, the general shape of the developed model was kept similar to that in this model. This model considers the axial influence of FRP bars by assuming a reduction factor for the tensile strength of FRP rebars. Although including the fractal model concept during the modeling can give more accurate results [53] but the fractal model concept of FRP bars (curvy geometrical figure and initial geometric imperfection) in the present study has been neglected to make the proposed model simple for the practical applications and to avoid the complexity of the model. The general shape of the projected theoretical model is reported by Equation (4).

(4) $$P_{n}=\alpha_{1}\left(A_{g}-A_{FRP}\right)f_{c}^{\prime }+\alpha_{2}f_{FRP}A_{FRP}$$

In this expression, $\alpha_{1}$ and $\alpha_{2}$ represent the reduction coefficients for the AS of FRP-reinforced compression members due to compressive influence of FRP rebars and confined core, $A_{FRP}$ is the area of FRP rebars, $A_{g}$ is the gross area of the column, $f_{FRP}$ is the tensile strength of FRP rebars. In the present investigation, the curve fitting method in MATLAB has been employed for securing the finest fit with the testing outcomes. The relationship for $\alpha_{1}$ as recommended by Ref. [11] can be reported by Equation (5).

(5) $$\alpha_{1}=0.85-\beta f_{c}^{\prime }$$

where β is another constant. Putting this value to Equation (6), the following relationship is obtained:

(6) $$P_{n}=\left(0.85-\beta f_{c}^{\prime }\right)\left(A_{g}-A_{FRP}\right)f_{c}^{\prime }+\alpha_{2}f_{FRP}A_{FRP}$$

**Figure 3:**
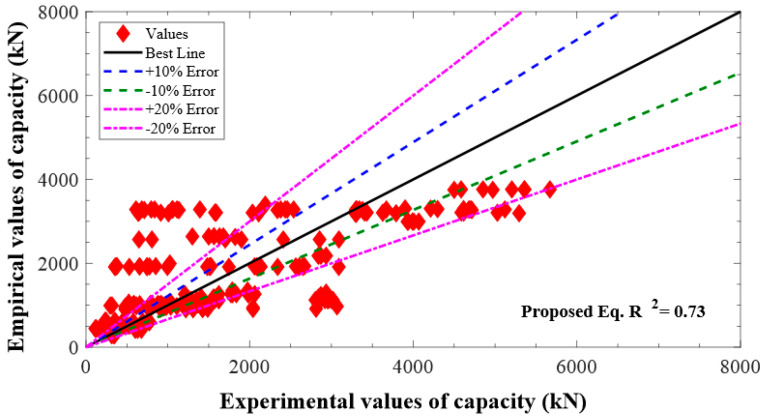
Performance of the suggested model.

The obtained values from the curve fitting method in MATLAB for the constants $\alpha_{2}$ and β were 0.0208 and 0.0029, correspondingly. Finally, the proposed model for the AS of FRP-reinforced members, after incorporating the values of the coefficients, has been reported by Equation (7).

(7) $$P_{n}=\left(0.85-0.0029f_{c}^{\prime }\right)\left(A_{g}-A_{FRP}\right)f_{c}^{\prime }+0.0208f_{FRP}A_{FRP}$$

where the reduction coefficient for AS of columns due to strength of concrete should be larger than 0.646 i.e., $\alpha_{1}=0.85-0.0029f_{c}^{\prime }\geq 0.646$. Figure 3 depicts that the newly suggested model for the AS of columns reported higher accuracy than the previous models with R^2^ = 0.73.

**Figure 4:**
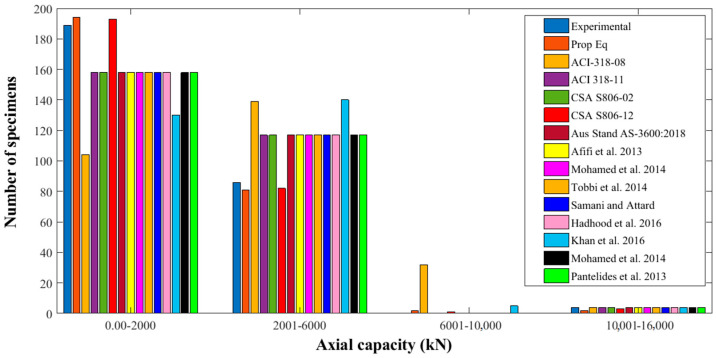
Distribution of AS of fiber-reinforced polymers (FRP)-reinforced columns attained from various models.

Figure 4 reports the sample circulation of the previous testing and predicted AS of FRP-reinforced columns. In range 0–2000 kN, the experimental datapoints were counted to be 189 in the developed database. There were 194 datapoints of the predictions of the suggested model. In the range, 2001–6000 kN, the experimental and theoretical counts were 81 and 86, correspondingly. Similarly, in the range, 6001–10,000 kN, the experimental and theoretical counts were zero and 2, correspondingly. Similarly, in the range, 10,001–16,000 kN, the experimental and theoretical counts were 4 and 2, correspondingly. These comparisons show that the predicted values of the proposed model apprehended the AS of FRP-reinforced columns very well.

**Figure 5:**
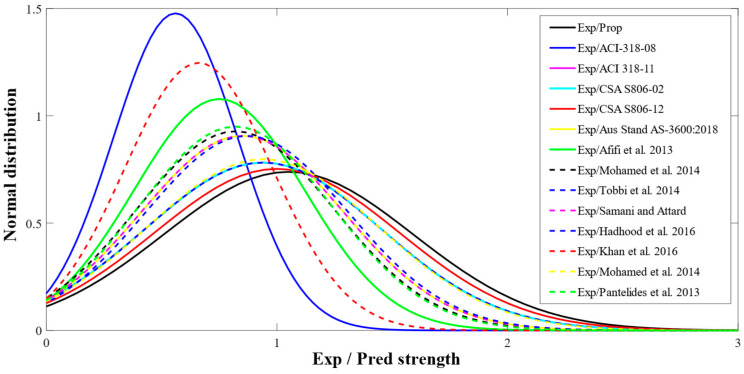
Normal distribution of testing to estimated strengths of GFRP-reinforced columns attained from various models.

Figure 5 reports the normal distribution of testing strengths to prophesied strengths of GFRP-reinforced compressive members from the created database for all models. The data of this figure has been obtained from the normalized predictions of various previous models over the developed database. The newly suggested model reported a deviation of only 5% for the average normalized strengths of the ratios of testing values to theoretical values. ACI-318-08 [44] reported a maximum deviation of 42%. Such errors may be ascribed to the cause that the model anticipated by ACI-318-08 [44] is for the steel rebars. This model has been used in this investigation for only a comparative purpose. Furthermore, the percent average eccentricities for the equations recommended by Afifi et al. [23], Khan et al. [47], CSA S806-12 [11] were 22%, 31%, and 5%, correspondingly.

## 4. Finite Element Modelling

**Figure 6:**
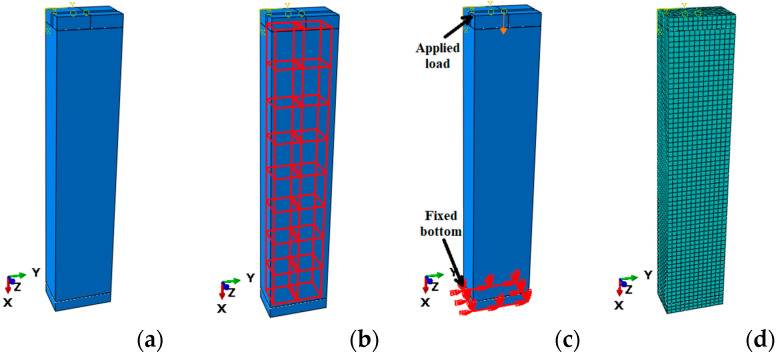
Finite element simulations of (**a**) geometry (**b**) interactions (**c**) support conditions (**d**) meshing of GFRP-RC specimens.

This section presents the methodology of the FEA of GFRP-RC columns under various loading situations. A total of seven GFRP-RC specimens were defined using a finite element software ABAQUS whose experimental results were taken from the previous research of Elchalakani et al. [29]. The details of all specimen are provided in Table 3. A control model (G150-45) was selected for the calibration purpose of the GFRP-RC columns. During the calibration of the control specimen, various geometric and materials characteristics of the control specimen such as support conditions, the plastic performance of concrete, element sizes, and various element types were studied to gain the results that give the minimum error as compared with the experimental outcomes. The boundary conditions were applied to the control model such that the bottom end of the specimen was controlled in all directions and the top portion of the specimen could freely translate and rotate in all directions. The simulations of GFRP reinforcement and concrete material were done using three-dimensional 8-noded brick and three-dimensional 2-noded truss elements, correspondingly. The bond behavior between the reinforcement and concrete was simulated using the “embedded region” that joins the degrees of freedom (DOF) of the truss sections of FRP bars to the essential DOF of concrete three-dimensional stress elements. The load was applied to the top center of the specimen using the displacement control technique. The geometry and support conditions of the simulated specimens are presented in Figure 6.

### 4.1. Simulation of Concrete Material

The behavior of concrete is complex due to the various constituents required for its manufacturing. The finite element simulation of this complex-natured material is a challenging task. In the present numerical work, the geometric performance of concrete was defined using three-dimensional brick elements with 8 nodes with reduced integration property (C3D8R). The average compressive strength of concrete material was 32 MPa at 28 days. The elastic behavior of concrete was defined using the equation given by ACI 318-11 [50] as presented by Equation (8).

(8) $$E_{c}=4700\sqrt{f_{c}^{\prime }}$$

**Figure 7:**
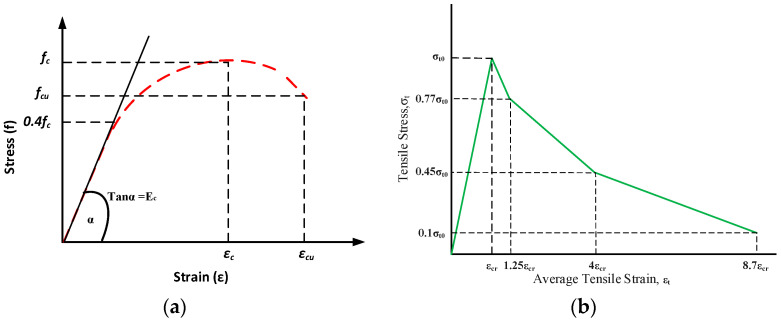
(**a**) Stress–strain relationship for concrete structures. (**b**) Tension stiffening model for concrete.

The concrete damaged plastic (CDP) model available in ABAQUS was used for the definition of the plastic performance of concrete. This relationship considers the crushing of concrete under compressive loading and the cracking of concrete under tensile loading to accurately predict the plastic behavior of concrete [54,55]. The CDP model divides the plasticity behavior of concrete into three parts: plastic, compressive, and tensile behavior. The plastic performance of concrete material was calibrated for all the parameters of plasticity available in the CDP model of concrete i.e., stress ratio, dilation angle, shape factor, viscosity parameter, and eccentricity of concrete. For the definition of concrete performance under compressive loading, the stress–strain relationship provided by Eurocode 2 [56] was utilized as presented by Figure 7a. The linear elastic behavior of concrete was taken up to 40% of the ultimate strength of concrete [57]. The ultimate strain ($\varepsilon_{cu}$) and the strain at ultimate compressive strength of concrete ($\varepsilon_{c}$) were calculated using Equation (9) and (10) as recommended by [58]. The compressive stresses ($\sigma_{c}$) were calculated using the relationship given by Eurocode 2 [56] as presented by Equation (10).

(9) $$\varepsilon_{c}=0.0014\left[2-e^{-0.024f_{c}}-e^{-0.140f_{c}}\right]$$

(10) $$\varepsilon_{cu\;}=0.004-0.0011\left[1-e^{-0.0215f_{c}}\right]$$

(11) $$\sigma_{c}=f_{c}\frac{k\eta -\eta^{2}}{1+\left(k-2\right)\eta }$$

where $k=1.05E_{c}\frac{\varepsilon_{cu}}{f_{c}},\;\eta =\;\frac{\varepsilon_{c}}{\varepsilon_{cu}}$.

The tensile behavior of concrete in the CDP model was defined using the modified tension stiffening model [59] as shown in Figure 7b. This model considers the behavior of concrete at post failure conditions of concrete such as tension stiffening, strain hardening, and softening and the interactions of FRP reinforcement with the concrete material. The tensile strength of concrete ($f_{t}^{\prime }$) was determined using the model proposed by Ref. [60].

(12) $$f_{t}^{\prime }=0.33\sqrt{f_{c}^{\prime }}$$

### 4.2. Simulations of FRP Bars

**Figure 8:**
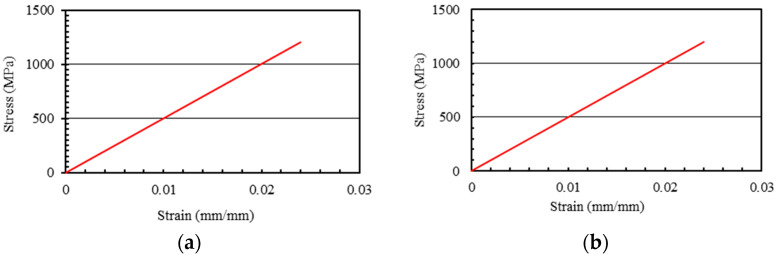
(**a**) Linear elastic performance of GFRP ties. (**b**) Linear elastic performance of GFRP longitudinal bars.

The geometric definitions of reinforcing bars were accomplished using 3-D truss sections having two nodes with three DOF at each node (T3D2). The definition of elastic performance of FRP bars was carried out using two variables i.e., Poisson’s ratio and Young’s modulus that were taken as 0.25 [37] and 50 GPa, correspondingly [29]. FRP bars show sudden failure with rupture after yielding strength. Therefore, the plastic performance of GFRP bars was assumed as linear elastic up to failure without the application of any damaging criterion [38]. The tensile strengths of longitudinal and transverse GFRP bars were considered as 1200 MPa and 784 MPa, correspondingly [29]. Figure 8 represents the simulated behavior of GFRP longitudinal and transverse bars in ABAQUS.

### 4.3. Calibration of FEM

One of the GFRP-RC columns (G150-45) was selected for calibration purposes. The numerical results of the load-deflection curve of the control model were compared with the experimental results from Ref. [29]. After calibrating the control model, it was used for the analysis of the other six specimens to further authenticate the accuracy of the anticipated finite element model. The control model was calibrated for different element types of GFRP and concrete material, mesh sizes, the eccentricity of concrete, shape factor, viscosity parameter, stress ratio, and dilation angle of concrete. 

**Figure 9:**
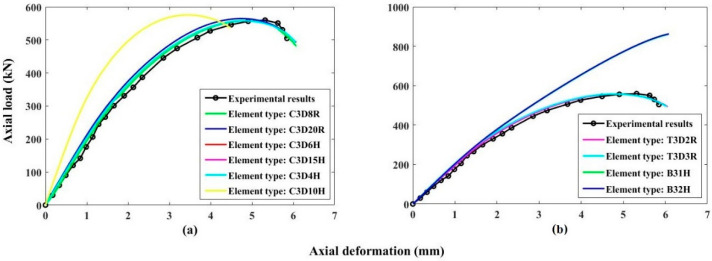
Sensitivity of various element types on load-deflection behavior of control specimen (**a**) concrete elements (**b**) FRP bars elements.

Various element types of concrete material and FRP reinforcement were evaluated to examine the effect of their variation on the load-deflection behavior of control finite element specimens. The 3-D stress elements available in the ABAQUS library for concrete material include hexahedral (C3D8R & C3D20R), tetrahedral (C3D4H & C3D10H), and triangular (C3D6H & C3D15H) elements that were studied during the calibration process. Similarly, the FRP reinforcing bars were studied for different truss (T3D2R & T3D3R) and beam (B31H & B32H) elements. It was observed that C3D8R and T3D2R gave the best results for concrete and reinforcement, correspondingly as presented in Figure 9 which displays the load-deflection response of the control column.

**Figure 10:**
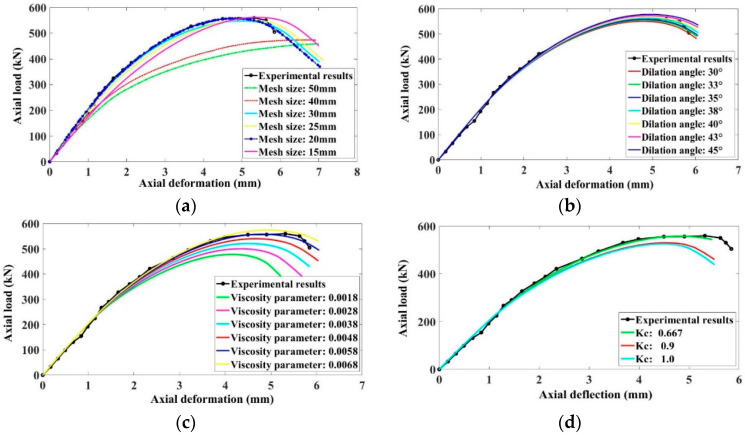
Sensitivity analysis of (**a**) mesh size (**b**) dilation angle (**c**) viscosity parameter (**d**) shape factor of concrete on the load-deflection curve of the control specimen.

The effect of increasing or decreasing the mesh size was also studied. The finite element models are always meshed size-dependent. This may be due to the phenomenon of strain localization that causes the unloading of some elements in the model. The studied mesh sizes were 15, 20, 25, 30, 40, and 50 mm. The best results were obtained while using a mesh size of 20 mm throughout the specimen. Figure 10a presents the load-deflection response of the control model by using different values of mesh size of the specimen.

The dilation angle of concrete, which represents the internal frictional angle, should range between 30° and 45° [61,62,63,64,65,66]. In the present investigation, the studied values of dilation angle were 30°, 33°, 35°, 38°, 40°, 43°, and 45°, correspondingly. The effect of variation of the dilation angle was not significant for the load-deflection response of the control specimen as reported in Figure 10b. However, the dilation angle of 35° gave a close agreement of numerical outcomes of the load-deflection curve with the experimental measurements.

The sensitivity of the viscosity parameter of concrete on the axial performance of the GFRP-RC column was also investigated. Various values of viscosity parameter used for the adjustment were 0.0068, 0.0058, 0.0048, 0.0038, 0.0028, and 0.0018. The effect of the viscosity parameter on the load-deflection response of the control model is presented in Figure 10c. There was an increase of 20.41% in the axial capacity of the specimen when the viscosity parameter was improved from 0.0018 to 0.0068. However, a close correlation with the experimental results was observed while using a viscosity parameter of 0.0058.

The effect of the shape factor for the yielding surface of the concrete on the load-deflection performance of the control model is presented in Figure 10d. With the increase or decrease of shape factor from 2/3, the ultimate load of the specimen decreases. Thus, the best approximation for the testing load-deflection behavior of the GFRP-RC control model was observed at a value of 2/3. Similarly, the effects of stress ratio and eccentricity of concrete were also examined. The results indicated that these two parameters have no significant effect on the axial performance of GFRP-RC specimens. Therefore, the default values i.e., 1.16 and 0.1 were used for these parameters, correspondingly.

## 5. Results and Discussion

The load-deflection curve of the control model represents that the percentage discrepancy between the numerical and experiments was 6.23% for the maximum loading capacity and 9.70% for the axial deflection at a maximum loading capacity of GFRP-RC specimen. However, the overall performance of the curve was closely captured by the proposed FEM. The minor discrepancies between the experimental and FEM results may be due to the differences between assumed boundary conditions in numerical simulations and experimental testing. Furthermore, the discrepancies may be associated with the geometric imperfections, differences in the strength of concrete and FRP material, the accuracy of laboratory instruments, manufacturing faults of specimens, and the assumptions made during the simulations.

After the validation of the control model, it was employed for the analysis of all other GFRP-RC columns from Ref. [29]. Table 4 represents the results obtained from the finite element simulations and their discrepancies from the experimental measurements. The average percentage discrepancy of finite element simulations from the experiments was 3.78% for loading capacity and 15.9% for the corresponding deflection at ultimate loading capacity.

### Load-Deflection Performance of FEM

**Figure 11:**
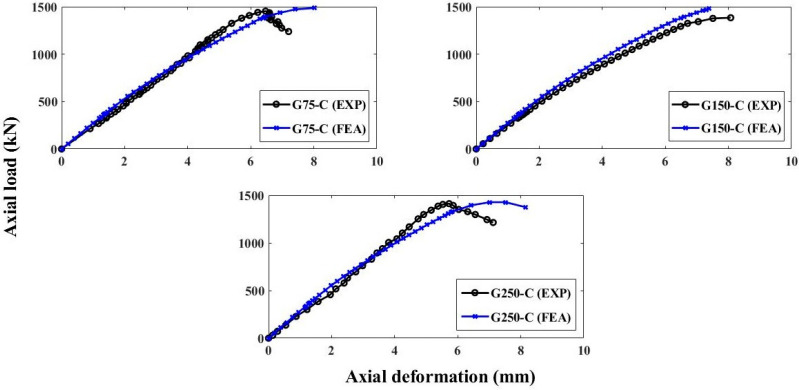
Load-deflection performance of GFRP-RC concentric columns.

The load-deflection curves of concentrically loaded GFRP-RC specimens are presented in Figure 11. The anticipated FEM predicted the axial behavior of concentric specimens with high accuracy in the elastic region of the load-deflection curve. However, the post-buckling behavior of specimens was not exactly traced. This may be due to the assumption of linear elastic performance of GFRP reinforcement up to failure in the simulations. In concentric columns, the GFRP bars are subjected to pure compression and the compressive performance of GFRP bars was taken as similar to the tensile behavior that may also be a reason for the discrepancy of results during the post-peak behavior. The FEM of the GFRP-RC specimen with 75 mm stirrup spacing tested under concentric loading (G75-C) portrayed the percentage faults of 2.56% and 13.92% for the maximum capacity and axial deflection at that capacity, correspondingly. The specimen G150-C showed percentage errors of 1.26% for axial capacity and 17.46% for the corresponding axial deflection. Similarly, the discrepancies for AS and axial deflections were 1.79% and 21.07%, correspondingly. The average differences between the simulations and experimental measurements of concentric GFRP-RCC specimens were 5.21% and 14.70% for peak loading capacity and axial deflection at that loading, correspondingly.

**Figure 12:**
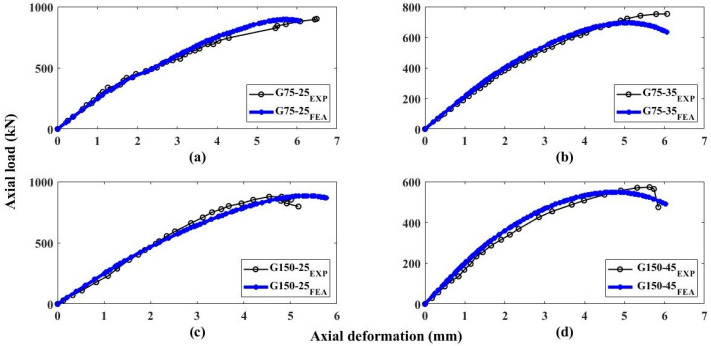
Load-deflection performance of GFRP-RC eccentric columns (**a**) G75-25 (**b**) G75-35 (**c**) G150-25 (**d**) G150-45.

In the case of eccentric GFRP-RC columns, the proposed FEM predicted the axial performance of specimens with high accuracy. Figure 12 represents the finite element and experimental load-deflection curves of the eccentrically loaded GFRP-RC specimens. It can be observed that the FEM of the specimen G75-25 gave a percentage error of 2.34% and 21.36% for the axial ultimate load and equivalent axial deflection, correspondingly. The percent error for specimen G75-35 was 11.84% for axial load and 17.45% for the axial deflection of the columns. The column with 150 mm stirrups spacing tested with an eccentricity of 25 mm (G150-25) presented the discrepancies of 0.41% and 10.28% for axial capacity and corresponding deflection, correspondingly. Similarly, the control specimen G150-45 presented the errors of only 6.23% and 9.70% for load and deflection, correspondingly. All the eccentrically loaded specimens showed an average discrepancy of 5.21% for the peak load and an average discrepancy of 14.70% for the axial deflection at the peak load. This discussion represents that the anticipated FEM predicts the axial loads of concentric GFRP-RC columns and axial deflections of eccentric GFRP-RC columns with higher accuracy.

## 6. Parametric Investigation

After validation, the anticipated FEM and empirical models were employed for the further analysis of 600 GFRP-reinforced rectangular columns under compressive loads. Four (4) different parameters of columns (a) longitudinal FRP reinforcement ratio ($\rho_{l}$), (b) concrete compressive strength ($f_{c}^{\prime }$), the tensile strength of GFRP rebars ($f_{u}$), and width of column (B) was varied for different ranges to examine their effect on the axial capacity (strength) of the columns as reported in Table 5. The main aim of the parametric study was to construct a database of GFRP-RC columns with various geometrical and material parameters so that the predictions of proposed capacity models could be verified and compared by using these results.

### 6.1. Using FEA Model

#### 6.1.1. Influence of Width of Column (B)

**Figure 13:**
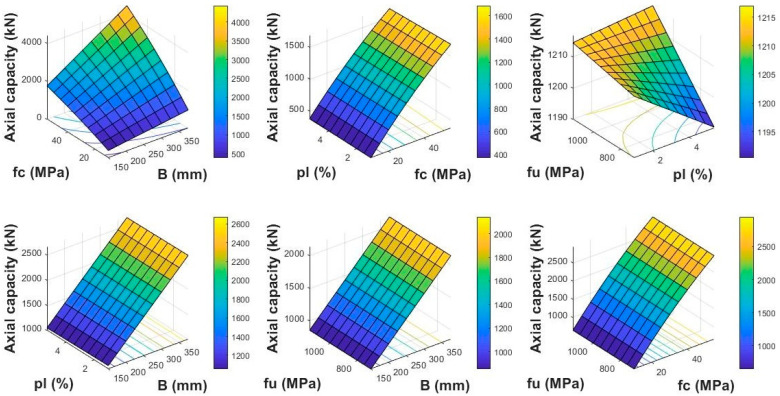
Detailed parametric investigation using FEM.

Figure 13 reports the influence of the effect of “B” of the GFRP-reinforced column on their AS. The examined values of this parameter were 375, 350, 325, 300, 275, 250, 225, 200, 175, and 150 mm. The enhancement of “B” from 150 to 375 mm resulted in an increase of 1041% in the axial compressive strength of GFRP-reinforced members with the enhancement of $f_{c}^{\prime }$ from 10 to 55 MPa at $\rho_{l}$ of 1.94% and fu of 850 MPa as fixed. Similarly, the enhancement of “B” from 150 to 375 mm resulted in an upsurge of 151% in the AS with the enhancement of $f_{u}$ from 700 to 1150 MPa.

#### 6.1.2. Influence of Concrete Compressive Strength ($f_{c}^{\prime }$)

Figure 13 reports the influence of $f_{c}^{\prime }$ on the axial compressive strength of columns. The upsurge of $f_{c}^{\prime }$ from 10 to 55 MPa resulted in an enhancement of 1041% in the AS with the increase of “B” from 150 to 375 mm. Similarly, the enhancement of $f_{c}^{\prime }$ with the increase of $\rho_{l}$ from 0.97% to 5.35% resulted in an improvement of 343% in the AS of columns. By enhancing $f_{c}^{\prime }$ from 10 to 55 MPa, the AS resulted in an improvement of 357% with the upsurge of $f_{u}$ from 700 to 1150 MPa. This portrays that the enhancement of tensile strength of FRP rebars and compressive strength of concrete results in a similar effect on the AS of FRP-reinforced concrete columns. 

#### 6.1.3. Influence of Longitudinal Reinforcement Ratio ($\rho_{l}$)

The influence of reinforcement ratio was also reported in Figure 13. This parameter has been examined for the various values including 5.35%, 4.86%, 4.38%, 3.89%, 3.41%, 2.92%, 2.43%, 1.94%, 1.46%, and 0.97%. The enhancement of $\rho_{l}$ from 0.97% to 5.35% resulted in an improvement of 343% in the AS with an upsurge of $f_{c}^{\prime }$ from 10 to 55 MPa. Similarly, the enhancement of $\rho_{l}$ with the upsurge of fu from 700 to 1150 MPa resulted in an enhancement of only 0.6%. Furthermore, enhancing the “B” from 150 to 375 mm resulted in an improvement of 149% in the AS with the enhancement of $\rho_{l}$ from 0.97% to 5.35%. 

#### 6.1.4. Influence of Tensile Strength of FRP Rebars ($f_{u}$)

The influence of changing the $f_{u}$ on the AS is reported in Figure 13. Different values of $f_{u}$ with an augmentation of 50 MPa was examined in the range of 700 to 1150 MPa. The enhancement of f_u_ from 700 to 1150 MPa resulted in an improvement of only 0.6% in the axial compressive strength with the increase of $\rho_{l}$ from 0.97% to 5.35%. An improvement of 151% was observed by enhancing “B” from 150 to 375 mm. Similarly, the enhancement of $f_{u}$ from 700 to 1150 MPa resulted in an improvement of only 357% in the axial compressive strength with the upsurge of $f_{c}^{\prime }$ from 10 to 55 MPa. Finally, it was concluded that the influence of the area of column and strength of concrete was significant on its axial compressive strength as compared with the other parameters.

### 6.2. Using Empirical Model

#### 6.2.1. Influence of Width of Column (B)

**Figure 14:**
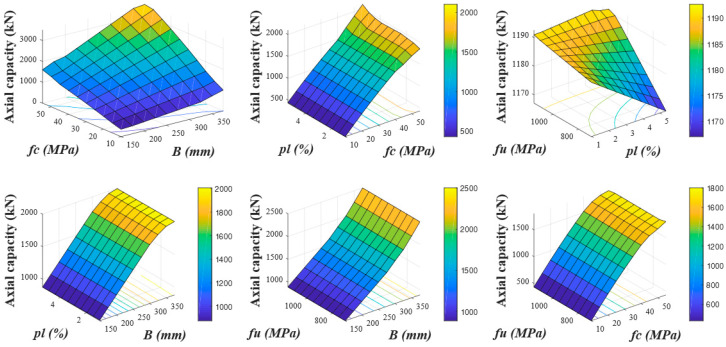
Parametric investigation using an empirical model.

The same parameters were evaluated in the parametric investigation using the proposed empirical model as shown in Figure 14 that displays the influence of “B” of GFRP-reinforced column on their AS. The examined values of this parameter were the same as in the numerical parameter study. The enhancement of “B” from 150 to 375 mm resulted in an increase of 1123% in the axial compressive strength of specimens with the enhancement of $f_{c}^{\prime }$ from 10 to 55 MPa at $\rho_{l}$ of 1.94% and f_u_ of 850 MPa as fixed. Similarly, the enhancement of “B” from 150 to 375 mm resulted in an upsurge of 214% in the AS with the enhancement of $f_{u}$ from 700 to 1150 MPa.

#### 6.2.2. Influence of Concrete Compressive Strength ($f_{c}^{\prime }$)

The effect of $f_{c}^{\prime }$ on the axial compressive strength of columns is reported in Figure 14. The upsurge of $f_{c}^{\prime }$ from 10 to 55 MPa resulted in an augmentation of 1123% in the AS with the increase of “B” from 150 to 375 mm. Similarly, the enhancement of $f_{c}^{\prime }$ with the increase of $\rho_{l}$ from 0.97% to 5.35% resulted in an upgrading of 389% in the AS of columns. By enhancing $f_{c}^{\prime }$ from 10 MPa to 55 MPa, the AS resulted in an improvement of 469% with the upsurge of f_u_ from 700 to 1150 MPa. 

#### 6.2.3. Influence of Longitudinal Reinforcement Ratio ($\rho_{l}$)

Figure 14 also presents the influence of the FRP reinforcement ratio ($\rho_{l}$) on the axial capacity of specimens. This parameter has been examined for the various values as investigated in the numerical parametric study. The enhancement of $\rho_{l}$ from 0.97% to 5.35% resulted in an improvement of 389% in the AS with an upsurge of $f_{c}^{\prime }$ from 10 to 55 MPa. Similarly, the enhancement of $\rho_{l}$ with the upsurge of fu from 700 to 1150 MPa resulted in an enhancement of 125%. Furthermore, enhancing the “B” from 150 to 375 mm resulted in an improvement of 236% in the AS with the enhancement of $\rho_{l}$ from 0.97% to 5.35%.

#### 6.2.4. Influence of Tensile Strength of FRP Rebars (f_u_)

The influence of changing the $f_{u}$ on the AS was reported in Figure 14. Different values of $f_{u}$ with an increase of 50 MPa were assessed in the range of 700–1150 MPa. The enhancement of $f_{u}$ from 700 to 1150 MPa resulted in an improvement of 125% in the axial compressive strength with the increase of $\rho_{l}$ from 0.97% to 5.35%. An improvement of 151% was observed by enhancing “B” from 150 to 375 mm. Similarly, the enhancement of $f_{u}$ from 700 to 1150 MPa resulted in an improvement of only 469% in the axial compressive strength with the upsurge of $f_{c}^{\prime }$ from 10 to 55 MPa. Therefore, it is concluded that during the parametric investigation, both the models (FEM and empirical models) have portrayed their predictions of the axial strength of GFRP-reinforced columns close to each other.

## 7. Validation and Comparison of Proposed Models

**Figure 15:**
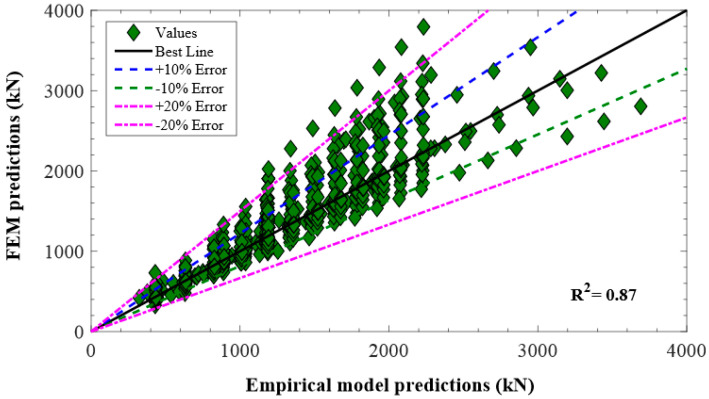
Comparison of predictions of anticipated empirical and FEM.

The purpose of the parametric study using the proposed FEM and empirical models in the present work was to generate a database of GFRP-reinforced compressive members to validate and compare the anticipated models for the axial compressive strength of GFRP-reinforced columns. The numerical and empirical database consisted of 600 results of AS of GFRP-reinforced concentric specimens. The theoretical predictions of these 600 GFRP-RC specimens calculated using the anticipated empirical model were compared with that of the numerical model. The comparative study of FEM and theoretical predictions are reported in Figure 15. It was noticed that the anticipated empirical axial capacity model performed well for the numerical parametric results with an R^2^ of 0.87. Thus, the anticipated empirical and FEM model is accurate enough to capture the AS of GFRP-reinforced concrete columns.

## 8. Conclusions

In the present work, an experimental database of 278 FRP-RC compression members was established from the literature to recommend an empirical model that can accurately predict the AS of GFRP-RC specimens. An initial evaluation of 13 different previously anticipated empirical models was executed to achieve a general shape of the AS model. Finally, a new empirical equation for forecasting the AS of GFRP-reinforced short columns was proposed using the curve fitting and regression analysis technique. A validated FEM was suggested for GFRP-reinforced members and used along with an empirical model for a detailed parametric investigation. Following conclusions were extracted from this work.

A better performance has been reported by the newly developed empirical model for apprehending the axial compressive strength of FRP-reinforced concrete compressive members with R^2^ = 0.73 using a database of 278 experimental data points. This model depicted higher correctness as compared with all previous models for different statistical errors (RMSE and MAE). Furthermore, the new model has been proposed based on a large data of FRP-reinforced columns while the previously proposed models were proposed using small databases.

The axial contribution of FRP rebars has been involved in the model with a reduction factor of $0.85-0.0029f_{c}^{\prime }\geq 0.646$ for the concrete strength and a reduction factor of 0.0208 for the AS of FRP rebars.

The suggested FEM also apprehended the structural performance of FRP-reinforced concrete members with a high accuracy depicting only the errors of 3.78% and 15.9% for AS and equivalent axial deflection, correspondingly.

The parametric investigation depicted that the AS of the concrete and cross-sectional area of the concrete column had a significant influence on the AS of such members. The enhancement of the cross-sectional area of the column by an increase of 1.5 times resulted in the AS up to 1041%. Correspondingly, the enhancement of concrete strength by 4.5 times resulted in an improvement of 357% in the AS of columns. The influence of reinforcement ratio of FRP rebars and tensile strength of FRP rebars reported no significant influence on the AS. 

Six hundred (600) specimens were tested in ABAQUS and empirical model to perform the parametric investigation and to generate a theoretical database for the assessment purposed FEM with the estimates of the theoretical model. The comparison reported a close agreement among each other with R^2^ = 0.87. Thus, the anticipated theoretical and FEA models are precise enough to further analyze the FRP-reinforced concrete columns.

## Figures and Tables

**Table 1:** Statistical details of different parameters of database.

Parameter	*B* (mm)	*H* (mm)	$f_{c}^{\prime }$ (MPa)	*D* (mm)	*Ag* (mm^2^)	$f_{u}\;(\mathrm{MPa})$	$E_{f}\;(\mathrm{GPa})$	$\varepsilon_{u}$ (%)	$\rho_{l}$ (%)	*A_f_* (mm^2^)	$\rho_{t}$ (%)	$P_{n}\;(\mathrm{kN})$
MIN	150	150	20.0	150	17662	406	23.4	0.97	0.55	212.53	0.01	114
MAX	610	610	70.2	305	372100	1680	141	2.42	5.3	4051.60	5.3	15235
Mean	249	272	36.2	258	66289	1010	56.7	1.78	2.09	1214.58	1.38	1814
*SD	114	114	12.6	54	53039	339	25.1	0.39	1.06	764.62	1.06	1877
**COV	0.46	0.43	0.35	0.21	0.81	0.34	0.45	0.22	0.51	0.63	0.77	1.04

* Standard deviation, ** coefficient of variation.

**Table 2:** Axial strength (AS) models for assessment.

Code/Research	Proposed Model
ACI-318-08 [44]	$P_{n}=0.85f_{c}^{\prime }\left(A_{g}-A_{s}\right)+f_{y}A_{s}$
CSA S806-02 [45]	$P_{n}=0.85f_{c}^{\prime }\left(A_{g}-A_{FRP}\right)$
CSA S806-12 [11]	$P_{n}=\alpha_{1}f_{c}^{\prime }\left(A_{g}-A_{FRP}\right);\;\alpha_{1}=0.85-0.0015f_{c}^{\prime }\geq 0.67$
Afifi et al. [23]	$P_{n}=0.85f_{c}^{\prime }\left(A_{g}-A_{FRP}\right)+\alpha_{g}f_{FRP}A_{FRP}$; $\alpha_{g}=0.35$
Samani and Attard [46]	$P_{n}=0.85f_{c}^{\prime }\left(A_{g}-A_{FRP}\right)+0.0025E_{FRP}A_{FRP}$
Khan et al. [47]	$P_{n}=0.85f_{cc}^{\prime }\left(A_{g}-A_{GFRP}\right)+\alpha f_{GFRP}A_{GFRP};$ α=0.61
Tobbi et al. [48]	$P_{n}=0.85f_{c}^{\prime }\left(A_{g}-A_{FRP}\right)+\varepsilon_{co}E_{FRP}A_{FRP};\;\varepsilon_{co}=0.003$
Pantelides et al. [49]	$P_{n}=0.85f_{ccFRP}^{\prime }A_{c}+A_{FRP}\varepsilon_{cFRP}E_{FRP};\;\varepsilon_{cFRP}=0.003$
ACI 318-11 [50]	$P_{n}=0.85f_{c}^{\prime }\left(A_{g}-A_{s}\right)$
AS-3600:2018 [51]	$P_{n}=0.85f_{c}^{\prime }\left(A_{g}-A_{FRP}\right)+0.0025E_{FRP}A_{FRP}$
Mohamed et al. [32]	$P_{n}=0.90f_{c}^{\prime }\left(A_{g}-A_{FRP}\right)+\varepsilon_{fg}E_{FRP}A_{FRP}$; $\varepsilon_{fg}=0.002$
Hadhood et al. [52]	$P_{n}=\alpha_{1}f_{c}^{\prime }\left(A_{g}-A_{FRP}\right)+0.0035E_{FRP}A_{FRP};$ $\alpha_{1}=0.85-0.0015f_{c}^{\prime }$
Mohamed et al. [32]	$P_{n}=0.85f_{c}^{\prime }\left(A_{g}-A_{FRP}\right)+\varepsilon_{p}E_{FRP}A_{FRP};$ $\varepsilon_{p}=0.002$

**Table 3:** Specimens for numerical simulations.

Sample Label	Longitudinal Reinforcement		Transverse Reinforcement	Eccentricity (mm)
	GFRP Bars	Reinforcing Ratio (%)		
G150-45	6-ɸ12.7 mm	1.83	6.35 mm @ 150 mm c/c	45
G150-25	6-ɸ12.7 mm	1.83	6.35 mm @ 150 mm c/c	25
G150-C	6-ɸ12.7 mm	1.83	6.35 mm @ 150 mm c/c	0
G75-35	6-ɸ12.7 mm	1.83	6.35 mm @ 75 mm c/c	35
G75-25	6-ɸ12.7 mm	1.83	6.35 mm @ 75 mm c/c	25
G75-C	6-ɸ12.7 mm	1.83	6.35 mm @ 75 mm c/c	0
G250-C	6-ɸ12.7 mm	1.83	6.35 mm @ 250 mm c/c	0

**Table 4:** Testing and numerical simulation results.

Sample Label	Experimental Results		FEA Results from ABAQUS		% Difference in Peak Loads (KN)	% Difference in Vertical Def. at Peak Load (mm)
	Peak Load (KN)	Vertical Deformation at Peak Load (mm)	Peak Load (KN)	Vertical Deformation at Peak Load (mm)		
G150-45	584.21	5.67	547.81	5.12	6.23	9.70
G150-25	880.28	4.86	883.94	5.36	0.41	10.28
G150-C	1366.76	6.87	1384.09	8.07	1.26	17.46
G75-35	787.80	6.13	694.51	5.06	11.84	17.45
G75-25	917.16	7.30	895.68	5.74	2.34	21.36
G75-C	1449.06	6.39	1486.26	7.28	2.56	13.92
G250-C	1401.8	5.79	1426.91	7.01	1.79	21.07

**Table 5:** Ranges of the parameters during the parametric investigation.

Variable	Fixed Value	Studied Values
Concrete strength (MPa)	30	10, 15, 20, 25, 30, 35, 40, 45, 50, 55
Tensile strength (MPa)	850	700, 750, 800, 850, 900, 950, 1000, 1050, 1100, 1150
Side length of column (mm)	200	150, 175, 200, 225, 250, 275, 300, 325, 350, 375
Reinforcement ratio (%)	1.94	0.97, 1.46, 1.94, 2.43, 2.92, 3.41, 3.89, 4.38, 4.86, 5.35

## Data Availability

Some or all data will be available upon request from the corresponding author.

## Appendix A

**Table A1:** Constructed experimental database (GS = GFRP spirals, GH = GFRP hoops, SS = steel spirals, SH = steel hoops, N = no lateral reinforcement).

Sr. No.	Research Study	B	H	D	$f_{c}^{\prime }$	$f_{u}$	$E_{f}$	$\varepsilon_{u}$	Longitudinal Reinforcement		Transverse Reinforcement		Axial Strength
		(mm)	(mm)	(mm)	(MPa)	(MPa)	(GPa)	(%)	Bars	$\rho_{l}$ (%)	Type	$\rho_{t}$ (%)	(kN)
1	Afifi et al. [23]	-	-	300	20	934	55.4	1.56	8 No. 5	2.2	GS	1	2920
2	Afifi et al. [23]	-	-	300	20	934	55.4	1.56	4 No. 5	1.1	GS	1	2826
3	Afifi et al. [23]	-	-	300	20	934	55.4	1.56	12 No. 5	3.2	GS	1	2998
4	Afifi et al. [23]	-	-	300	20	934	55.4	1.56	8 No. 5	2.2	GS	0.45	2857
5	Afifi et al. [23]	-	-	300	20	934	55.4	1.56	8 No. 5	2.2	GS	1.87	3019
6	Afifi et al. [23]	-	-	300	20	934	55.4	1.56	8 No. 5	2.2	GS	2.07	2964
7	Afifi et al. [23]	-	-	300	20	934	55.4	1.56	8 No. 5	2.2	GS	0.69	2804
8	Afifi et al. [23]	-	-	300	20	934	55.4	1.56	8 No. 5	2.2	GS	1.03	2951
9	Afifi et al. [23]	-	-	300	20	934	55.4	1.56	8 No. 5	2.2	GS	1.03	2865
10	Afifi et al. [33]	-	-	301	21	934	55.4	1.56	8 No. 5	2.2	GS	1.5	2840
11	Afifi et al. [33]	-	-	302	22	934	55.4	1.56	8 No. 5	2.2	GS	1.5	2871
12	Afifi et al. [33]	-	-	303	23	934	55.4	1.56	8 No. 5	2.2	GS	1.5	2935
13	AlAjarmeh et al. [67]	-	-	250	31.8	1237	60	2.1	6 No. 5	2.41	GS	1.49	1588
14	AlAjarmeh et al. [67]	-	-	250	31.8	1237	60	2.1	6 No. 5	2.47	GS	1.56	1408
15	AlAjarmeh et al. [67]	-	-	250	31.8	1237	60	2.1	6 No. 5	2.59	GS	1.69	1559
16	AlAjarmeh et al. [67]	-	-	250	31.8	1237	60	2.1	6 No. 5	2.78	GS	1.92	1411
17	AlAjarmeh et al. [68]	-	-	251	25	1281.5	61.3	2.1	6 No. 4	1.78	GS	1.57	1035.3
18	AlAjarmeh et al. [68]	-	-	252	25	1237.4	60.5	2.1	6 No. 5	2.79	GS	1.57	1109.2
19	AlAjarmeh et al. [68]	-	-	253	25	1270	60.5	2.1	6 No. 6	4	GS	1.57	1247.9
20	AlAjarmeh et al. [68]	-	-	254	25	1237.4	60.5	2.1	4 No. 5	1.86	GS	1.57	983.3
21	AlAjarmeh et al. [68]	-	-	255	25	1237.4	60.5	2.1	8 No. 5	3.72	GS	1.57	1406.1
22	AlAjarmeh et al. [68]	-	-	256	25	1281.5	61.3	2.1	9 No. 4	2.67	GS	1.57	1204.2
23	Alsayed et al. [69]	250	450	-	39	800	40	1.5	6 No. 5	1	SH	0.15	3285
24	Alsayed et al. [69]	250	450	-	39	800	40	1.5	6 No. 5	1	SH	0.15	3285
25	Alsayed et al. [69]	250	450	-	39	800	40	1.5	6 No. 5	1	SH	0.15	3285
26	Alsayed et al. [69]	250	450	-	38.5	800	40	1.5	6 No. 5	1	GH	0.18	3301
27	Alsayed et al. [69]	250	450	-	38.5	800	40	1.5	6 No. 5	1	GH	0.18	3301
28	Alsayed et al. [69]	250	450	-	38.5	800	40	1.5	6 No. 5	1	GH	0.18	3301
29	De Luca et al. [70]	610	610	-	43.7	608	44.2	1.38	8 No. 8	1	GH	0.63	15235
30	De Luca et al. [70]	610	610	-	40.6	712	44.4	1.6	8 No 8	1	GH	0.63	12949
31	De Luca et al. [70]	610	610	-	36.1	608	44.2	1.38	8 No. 8	1	GH	2.5	11926
32	De Luca et al. [70]	610	610	-	32.8	712	44.4	1.6	8 No 8	1	GH	2.5	10751
33	Dong et al. [15]	-	-	215	40	930	59	1.6	3 No. 3	0.55	GS	0.94	1018
34	Dong et al. [15]	-	-	215	40	930	59	1.6	4 No. 3	0.73	GS	0.94	1179
35	Dong et al. [15]	-	-	215	40	930	59	1.6	5 No. 3	0.92	GS	0.94	1288
36	Dong et al. [15]	-	-	215	40	930	59	1.6	6 No. 3	1.1	GS	0.94	1381
37	Dong et al. [15]	-	-	215	40	930	59	1.6	4 No. 3	0.73	GS	2.75	1459
38	Dong et al. [15]	-	-	215	40	930	59	1.6	4 No. 3	0.73	GS	2.75	1037
39	Dong et al. [15]	-	-	215	40	880	59	1.6	4 No. 3	0.73	GS	2.75	523
40	Dong et al. [15]	-	-	215	37	880	59	1.6	4 No. 3	0.73	GS	2.75	318
41	Dong et al. [15]	-	-	215	37	880	59	1.6	5 No. 3	0.73	GS	1.39	1290
42	Dong et al. [15]	-	-	215	37	880	59	1.6	6 No. 3	0.73	GS	1.39	944
43	Dong et al. [15]	-	-	215	37	880	59	1.6	7 No. 3	0.73	GS	1.39	527
44	Dong et al. [15]	-	-	215	37	880	59	1.6	8 No. 3	0.73	GS	1.39	296
45	Elchalakani and Ma [26]	160	260	-	32.8	1200	50	2.4	6 No. 4	1.8	GH	0.5	1367
46	Elchalakani and Ma [26]	160	260	-	32.8	1200	50	2.4	6 No. 4	1.8	GH	0.5	880
47	Elchalakani and Ma [26]	160	260	-	32.8	1200	50	2.4	6 No. 4	1.8	GH	0.5	584
48	Elchalakani and Ma [26]	160	260	-	32.8	1200	50	2.4	6 No. 4	1.8	GH	1	1449
49	Elchalakani and Ma [26]	160	260	-	32.8	1200	50	2.4	6 No. 4	1.8	GH	1	917
50	Elchalakani and Ma [26]	160	260	-	32.8	1200	50	2.4	6 No. 4	1.8	GH	1	788
51	Elchalakani and Ma [26]	160	260	-	32.8	1200	50	2.4	6 No. 4	1.8	GH	0.3	1402
52	Elchalakani et al. [29]	160	260	-	32.8	930	59	1.7	6 No. 4	1.8	GH	0.3	1402
53	Elchalakani et al. [29]	160	260	-	32.8	930	59	1.7	6 No. 4	1.8	GH	0.5	1367
54	Elchalakani et al. [29]	160	260	-	32.8	930	59	1.7	6 No. 4	1.8	GH	1	1449
55	Elchalakani et al. [29]	160	260	-	32.8	930	59	1.7	6 No. 4	1.8	GH	0.5	880
56	Elchalakani et al. [29]	160	260	-	32.8	930	59	1.7	6 No. 4	1.8	GH	1	917
57	Elchalakani et al. [29]	160	260	-	32.8	930	59	1.7	6 No. 4	1.8	GH	1	788
58	Elchalakani et al. [29]	160	260	-	32.8	930	59	1.7	6 No. 4	1.8	GH	0.5	584
59	Elchalakani et al. [29]	160	260	-	32.8	930	59	1.7	6 No. 4	1.8	GH	0.3	1041
60	Elchalakani et al. [29]	160	260	-	32.8	930	59	1.7	6 No. 4	1.8	GH	0.5	1194
61	Elchalakani et al. [29]	160	260	-	32.8	930	59	1.7	6 No. 4	1.8	GH	1	1357
62	Elchalakani et al. [29]	160	260	-	32.8	930	59	1.7	6 No. 4	1.8	GH	0.5	657
63	Elchalakani et al. [29]	160	260	-	32.8	930	59	1.7	6 No. 4	1.8	GH	1	804
64	Elchalakani et al. [29]	160	160	-	32.8	930	59	1.7	6 No. 4	1.8	GH	0.5	353
65	Elchalakani et al. [29]	160	160	-	32.8	930	59	1.7	6 No. 4	1.8	GH	1	454
66	Elchalakani et al. [29]	160	160	-	32.8	930	59	1.7	6 No. 4	1.8	GH	0.5	234
67	Elchalakani et al. [29]	160	160	-	32.8	930	59	1.7	6 No. 4	1.8	GH	1	244
68	Guerin et al. [71]	405	405	-	25.3	600	40	1.5	6 No. 6	1	GH	0.66	4587
69	Guerin et al. [71]	405	405	-	25.3	600	40	1.5	6 No. 6	1	GH	0.66	3433
70	Guerin et al. [71]	405	405	-	25.3	600	40	1.5	6 No. 6	1	GH	0.66	1591
71	Guerin et al. [71]	405	405	-	25.3	600	40	1.5	6 No. 6	1	GH	0.66	645
72	Guerin et al. [71]	405	405	-	25.3	600	40	1.5	6 No. 6	1	GH	0.66	4616
73	Guerin et al. [71]	405	405	-	25.3	600	40	1.5	6 No. 6	1	GH	0.66	3405
74	Guerin et al. [71]	405	405	-	25.3	600	40	1.5	6 No. 6	1	GH	0.66	1576
75	Guerin et al. [71]	405	405	-	25.3	600	40	1.5	6 No. 6	1	GH	0.66	636
76	Guerin et al. [72]	405	405	-	25.3	600	40	1.5	8 No. 6	1.4	GH	0.84	5028
77	Guerin et al. [72]	405	405	-	25.3	600	40	1.5	8 No. 6	1.4	GH	0.84	3627
78	Guerin et al. [72]	405	405	-	25.3	600	40	1.5	8 No. 6	1.4	GH	0.84	2035
79	Guerin et al. [72]	405	405	-	25.3	600	40	1.5	8 No. 6	1.4	GH	0.84	914
80	Guerin et al. [72]	405	405	-	25.3	600	40	1.5	8 No. 8	2.5	GH	0.63	5294
81	Guerin et al. [72]	405	405	-	25.3	600	40	1.5	8 No. 8	2.5	GH	0.63	3790
82	Guerin et al. [72]	405	405	-	25.3	600	40	1.5	8 No. 8	2.5	GH	0.63	2110
83	Guerin et al. [72]	405	405	-	25.3	600	40	1.5	8 No. 8	2.5	GH	0.63	1008
84	Hadhood et al. [52]	-	-	305	35	1680	141	1.19	8 No. 5	2.2	GH	2.68	2564
85	Hadhood et al. [52]	-	-	305	35	1680	141	1.19	8 No. 5	2.2	GH	2.68	2060
86	Hadhood et al. [52]	-	-	305	35	1680	141	1.19	8 No. 5	2.2	GH	2.68	1511
87	Hadhood et al. [52]	-	-	305	35	1680	141	1.19	8 No. 5	2.2	GH	2.68	776
88	Hadhood et al. [52]	-	-	305	35	1680	141	1.19	8 No. 5	2.2	GH	2.68	366
89	Hadhood et al. [52]	-	-	305	35	1680	141	1.19	8 No. 5	2.2	GS	1	2608
90	Hadhood et al. [52]	-	-	305	35	1680	141	1.19	8 No. 5	2.2	GS	1	2134
91	Hadhood et al. [52]	-	-	305	35	1680	141	1.19	8 No. 5	2.2	GS	1	1513
92	Hadhood et al. [52]	-	-	305	35	1680	141	1.19	8 No. 5	2.2	GS	1	745
93	Hadhood et al. [52]	-	-	305	35	1680	141	1.19	8 No. 5	2.2	GS	1	654
94	Hadhood et al. [52]	-	-	305	35	1680	141	1.19	12 No. 5	3.3	GS	1	2670
95	Hadhood et al. [52]	-	-	305	35	1680	141	1.19	12 No. 5	3.3	GS	1	2123
96	Hadhood et al. [52]	-	-	305	35	1680	141	1.19	12 No. 5	3.3	GS	1	1527
97	Hadhood et al. [52]	-	-	305	35	1680	141	1.19	12 No. 5	3.3	GS	1	852
98	Hadhood et al. [52]	-	-	305	35	1680	141	1.19	12 No. 5	3.3	GS	1	378
99	Hadhood et al. [73]	-	-	305	35	1680	141	1.19	8 No. 5	2.2	GS	1.8	2652
100	Hadhood et al. [73]	-	-	305	35	1680	141	1.19	8 No. 5	2.2	GS	1.8	2086
101	Hadhood et al. [73]	-	-	305	35	1680	141	1.19	8 No. 5	2.2	GS	1.8	1483
102	Hadhood et al. [73]	-	-	305	35	1680	141	1.19	8 No. 5	2.2	GS	1.8	747
103	Hadhood et al. [73]	-	-	305	35	1680	141	1.19	8 No. 5	2.2	GS	1.8	655
104	Hadhood et al. [73]	-	-	305	70.2	1289	54.9	2.3	8 No. 5	2.2	GS	1.1	4709
105	Hadhood et al. [73]	-	-	305	70.2	1289	54.9	2.3	8 No. 5	2.2	GS	1.1	3309
106	Hadhood et al. [73]	-	-	305	70.2	1289	54.9	2.3	8 No. 5	2.2	GS	1.1	2380
107	Hadhood et al. [73]	-	-	305	70.2	1289	54.9	2.3	8 No. 5	2.2	GS	1.1	1112
108	Hadhood et al. [73]	-	-	305	70.2	1289	54.9	2.3	8 No. 5	2.2	GS	1.1	797
109	Hadhood et al. [73]	-	-	305	70.2	1289	54.9	2.3	8 No. 5	2.2	GH	1.1	4689
110	Hadhood et al. [73]	-	-	305	70.2	1289	54.9	2.3	8 No. 5	2.2	GH	1.1	3299
111	Hadhood et al. [73]	-	-	305	70.2	1289	54.9	2.3	8 No. 5	2.2	GH	1.1	2435
112	Hadhood et al. [73]	-	-	305	70.2	1289	54.9	2.3	8 No. 5	2.2	GH	1.1	1054
113	Hadhood et al. [73]	-	-	305	70.2	1289	54.9	2.3	8 No. 5	2.2	GH	1.1	838
114	Hadhood et al. [73]	-	-	305	70.2	1289	54.9	2.3	12 No. 5	3.2	GS	1.1	4716
115	Hadhood et al. [73]	-	-	305	70.2	1289	54.9	2.3	12 No. 5	3.2	GS	1.1	3380
116	Hadhood et al. [73]	-	-	305	70.2	1289	54.9	2.3	12 No. 5	3.2	GS	1.1	2339
117	Hadhood et al. [73]	-	-	305	70.2	1289	54.9	2.3	12 No. 5	3.2	GS	1.1	1135
118	Hadhood et al. [73]	-	-	305	70.2	1289	54.9	2.3	12 No. 5	3.2	GS	1.1	713
119	Hadhood et al. [73]	-	-	305	70.2	1289	54.9	2.3	8 No. 5	2.2	GS	1.1	5120
120	Hadhood et al. [73]	-	-	305	70.2	1289	54.9	2.3	8 No. 5	2.2	GS	1.1	3671
121	Hadhood et al. [73]	-	-	305	70.2	1289	54.9	2.3	8 No. 5	2.2	GS	1.1	2538
122	Hadhood et al. [73]	-	-	305	70.2	1289	54.9	2.3	8 No. 5	2.2	GS	1.1	1392
123	Hadhood et al. [73]	-	-	305	70.2	1289	54.9	2.3	8 No. 5	2.2	GS	1.1	611
124	Hadhood et al. [73]	-	-	305	70.2	1289	54.9	2.3	8 No. 5	2.2	GS	1.7	4680
125	Hadhood et al. [73]	-	-	305	70.2	1289	54.9	2.3	8 No. 5	2.2	GS	1.7	3341
126	Hadhood et al. [73]	-	-	305	70.2	1289	54.9	2.3	8 No. 5	2.2	GS	1.7	2460
127	Hadhood et al. [73]	-	-	305	70.2	1289	54.9	2.3	8 No. 5	2.2	GS	1.7	1061
128	Hadhood et al. [73]	-	-	305	70.2	1289	54.9	2.3	8 No. 5	2.2	GS	1.7	682
129	Hadhood et al. [73]	-	-	305	35	1289	54.9	2.3	8 No. 5	2.2	GS	1.1	2608
130	Hadhood et al. [73]	-	-	305	35	1289	54.9	2.3	8 No. 5	2.2	GS	1.1	2134
131	Hadhood et al. [73]	-	-	305	35	1289	54.9	2.3	8 No. 5	2.2	GS	1.1	1512
132	Hadhood et al. [73]	-	-	305	35	1289	54.9	2.3	8 No. 5	2.2	GS	1.1	745
133	Hadhood et al. [73]	-	-	305	35	1289	54.9	2.3	8 No. 5	2.2	GS	1.1	354
134	Hadhood et al. [73]	-	-	305	35	1289	54.9	2.3	8 No. 5	2.2	GS	1.1	3090
135	Hadhood et al. [73]	-	-	305	35	1289	54.9	2.3	8 No. 5	2.2	GS	1.1	2342
136	Hadhood et al. [73]	-	-	305	35	1289	54.9	2.3	8 No. 5	2.2	GS	1.1	1746
137	Hadhood et al. [73]	-	-	305	35	1289	54.9	2.3	8 No. 5	2.2	GS	1.1	995
138	Hadhood et al. [73]	-	-	305	35	1289	54.9	2.3	8 No. 5	2.2	GS	1.1	529
139	Hadhood et al. [73]	-	-	305	35	1289	54.9	2.3	8 No. 5	2.2	GS	1.1	2652
140	Hadhood et al. [73]	-	-	305	35	1289	54.9	2.3	8 No. 5	2.2	GS	1.1	2086
141	Hadhood et al. [73]	-	-	305	35	1289	54.9	2.3	8 No. 5	2.2	GS	1.1	1483
142	Hadhood et al. [73]	-	-	305	35	1289	54.9	2.3	8 No. 5	2.2	GS	1.1	747
143	Hadhood et al. [73]	-	-	305	35	1289	54.9	2.3	8 No. 5	2.2	GS	1.1	355
144	Hadi et al. [34]	-	-	205	37	1200	50	2.4	6 No. 4	1.6	GS	2.1	1220
145	Hadi et al. [34]	-	-	205	37	1200	50	2.4	6 No. 4	1.6	GS	2.1	781
146	Hadi et al. [34]	-	-	205	37	1200	50	2.4	6 No. 4	1.6	GS	2.1	494
147	Hadi et al. [34]	-	-	205	37	1200	50	2.4	6 No. 4	1.6	GS	4.2	1309
148	Hadi et al. [34]	-	-	205	37	1200	50	2.4	6 No. 4	1.6	GS	4.2	767
149	Hadi et al. [34]	-	-	205	37	1200	50	2.4	6 No. 4	1.6	GS	4.2	479
150	Hadi and Youssef [74]	210	210	-	29.3	1641	67.9	2.41	4 No. 4	1	GH	2.74	1285
151	Hadi and Youssef [74]	210	210	-	29.3	1641	67.9	2.41	4 No. 4	1	GH	2.74	803
152	Hadi and Youssef [74]	210	210	-	29.3	1641	67.9	2.41	4 No. 4	1	GH	2.74	615
153	Hassan et al. [75]	-	-	150	40	800	30	0.97	6 No. 3	2.1	SS	1.7	426.59
154	Hassan et al. [75]	-	-	150	40	800	30	1.35	6 No. 3	2.1	SS	1.7	411.88
155	Hassan et al. [75]	-	-	150	40	800	30	1.57	6 No. 3	2.1	SS	1.7	387.36
156	Hassan et al. [75]	-	-	150	40	800	30	1.4	6 No. 3	2.1	SS	3.4	529.56
157	Hassan et al. [75]	-	-	150	40	800	30	1.7	6 No. 3	2.1	SS	3.4	490.33
158	Hassan et al. [75]	-	-	150	40	800	30	1.9	6 No. 3	2.1	SS	3.4	460.91
159	Hassan et al. [75]	-	-	150	40	800	30	1.28	6 No. 3	2.1	GH	1.7	490.33
160	Hassan et al. [75]	-	-	150	40	800	30	1.5	6 No. 3	2.1	GH	1.7	460.91
161	Hassan et al. [75]	-	-	150	40	800	30	1.7	6 No. 3	2.1	GH	1.7	430.4
162	Karim et al. [35]	-	-	205	37	1600	66	2.42	6 No. 4	4.72	GS	1.91	1425
163	Karim et al. [35]	-	-	205	37	1600	66	2.42	6 No. 4	4.72	GS	3.82	2041
164	Karim et al. [52]	-	-	206	37	1600	66	2.42	6 No. 4	4.72	GS	1.91	1425
165	Karim et al. [52]	-	-	207	37	1600	66	2.42	6 No. 4	4.72	GS	1.91	781
166	Karim et al. [52]	-	-	208	37	1600	66	2.42	6 No. 4	4.72	GS	1.91	494
167	Karim et al. [52]	-	-	209	37	1600	66	2.42	6 No. 4	4.72	GS	3.82	2041
168	Karim et al. [52]	-	-	210	37	1600	66	2.42	6 No. 4	4.72	GS	3.82	767
169	Karim et al. [52]	-	-	211	37	1600	66	2.42	6 No. 4	4.72	GS	3.82	479
170	Karim et al. [52]	-	-	212	37	1600	66	2.42	6 No. 4	4.72	GS	1.91	3068
171	Karim et al. [52]	-	-	213	37	1600	66	2.42	6 No. 4	4.72	GS	1.91	1450
172	Karim et al. [52]	-	-	214	37	1600	66	2.42	6 No. 4	4.72	GS	1.91	805
173	Khan et al. [47]	-	-	206	37	1395	56	1.5	6 No. 5	3.57	GH	-	2812
174	Khan et al. [47]	-	-	206	37	1395	56	1.5	6 No. 5	3.57	GH	-	1487
175	Khan et al. [47]	-	-	206	37	1395	56	1.5	6 No. 5	3.57	GH	-	910
176	Khorramian & Sadeghian [27]	150	150	-	37	629	38.7	1.62	6 No. 5	5.3	N	-	775
177	Khorramian & Sadeghian [27]	150	150	-	37	629	38.7	1.62	6 No. 5	5.3	N	-	775
178	Khorramian & Sadeghian [27]	150	150	-	37	629	38.7	1.62	6 No. 5	5.3	N	-	693
179	Khorramian & Sadeghian [27]	150	150	-	37	629	38.7	1.62	6 No. 5	5.3	N	-	693
180	Khorramian & Sadeghian [27]	150	150	-	37	629	38.7	1.62	6 No. 5	5.3	N	-	693
181	Khorramian & Sadeghian [27]	150	150	-	37	629	38.7	1.62	6 No. 5	5.3	N	-	578
182	Khorramian & Sadeghian [27]	150	150	-	37	629	38.7	1.62	6 No. 5	5.3	N	-	578
183	Khorramian & Sadeghian [27]	150	150	-	37	629	38.7	1.62	6 No. 5	5.3	N	-	354
184	Khorramian & Sadeghian [27]	150	150	-	37	629	38.7	1.62	6 No. 5	5.3	N	-	354
185	Maranan et al. [76]	-	-	250	34.42	1184	62.6	1.89	6 No. 5	2.43	GH	3.13	1772
186	Maranan et al. [76]	-	-	250	34.42	1184	62.6	1.89	6 No. 5	2.43	GH	3.13	1791
187	Maranan et al. [76]	-	-	250	34.42	1184	62.6	1.89	6 No. 5	2.43	GH	1.57	1981
188	Maranan et al. [76]	-	-	250	34.42	1184	62.6	1.89	6 No. 5	2.43	GH	0.78	1988
189	Maranan et al. [76]	-	-	250	34.42	1184	62.6	1.89	6 No. 5	2.43	GS	3.13	1838
190	Maranan et al. [76]	-	-	250	34.42	1184	62.6	1.89	6 No. 5	2.43	GS	1.57	2063
191	Maranan et al. [76]	-	-	250	34.42	1184	62.6	1.89	6 No. 5	2.43	GH	1.57	1624
192	Maranan et al. [76]	-	-	250	34.42	1184	62.6	1.89	6 No. 5	2.43	GS	1.57	1208
193	Mohamed et al. [32]	-	-	300	42.9	934	55.4	1.56	8 No. 5	2.2	GH	2.23	2840
194	Mohamed et al. [32]	-	-	300	42.9	934	55.4	1.56	8 No. 5	2.2	GH	2.68	2871
195	Mohamed et al. [32]	-	-	300	42.9	934	55.4	1.56	8 No. 5	2.2	GH	3.14	2935
196	Pantelides et al. [49]	-	-	254	36	740	43.3	1.71	4 No. 5	1.6	GS	0.75	1975
197	Pantelides et al. [49]	-	-	254	36	740	43.3	1.71	4 No. 5	1.6	GS	0.75	1788
198	Prachasaree et al. [77]	150	150	-	20.8	735	50	1.5	4 No. 3	1.4	SS	0.01	370
199	Prachasaree et al. [77]	150	150	-	20.8	735	50	1.5	4 No. 3	1.4	SS	0.01	370
200	Prachasaree et al. [77]	150	150	-	20.8	735	50	1.5	4 No. 3	1.4	SS	0.01	370
201	Prachasaree et al. [77]	150	150	-	20.8	735	50	1.5	4 No. 3	1.4	SS	0.02	365
202	Prachasaree et al. [77]	150	150	-	20.8	735	50	1.5	4 No. 3	1.4	SS	0.02	365
203	Prachasaree et al. [77]	150	150	-	20.8	735	50	1.5	4 No. 3	1.4	SS	0.02	365
204	Prachasaree et al. [77]	-	-	150	20.8	735	50	1.5	4 No. 3	1.9	SS	0.01	345
205	Prachasaree et al. [77]	-	-	150	20.8	735	50	1.5	4 No. 3	1.9	SS	0.01	345
206	Prachasaree et al. [77]	-	-	150	20.8	735	50	1.5	4 No. 3	1.9	SS	0.01	345
207	Prachasaree et al. [77]	-	-	150	20.8	735	50	1.5	4 No. 3	1.9	SS	0.02	315
208	Prachasaree et al. [77]	-	-	150	20.8	735	50	1.5	4 No. 3	1.9	SS	0.02	315
209	Prachasaree et al. [77]	-	-	150	20.8	735	50	1.5	4 No. 3	1.9	SS	0.02	315
210	Prachasaree et al. [77]	150	150	-	20.8	735	50	1.5	4 No. 3	1.4	SH	0.01	365
211	Prachasaree et al. [77]	150	150	-	20.8	735	50	1.5	4 No. 3	1.4	SH	0.01	365
212	Prachasaree et al. [77]	150	150	-	20.8	735	50	1.5	4 No. 3	1.4	SH	0.01	365
213	Prachasaree et al. [77]	150	150	-	20.8	735	50	1.5	4 No. 3	1.4	SH	0.02	370
214	Prachasaree et al. [77]	150	150	-	20.8	735	50	1.5	4 No. 3	1.4	SH	0.02	370
215	Prachasaree et al. [77]	150	150	-	20.8	735	50	1.5	4 No. 3	1.4	SH	0.02	370
216	Sankholkar et al. [78]	-	-	203	50	800	46.2	1.57	4 No. 5	2.5	GS	3.2	1353
217	Sankholkar et al. [78]	-	-	203	50	800	46.2	1.57	4 No. 5	2.5	GS	3.2	1285
218	Sankholkar et al. [78]	-	-	203	50	800	46.2	1.57	6 No. 5	3.7	GS	3.2	1623
219	Sankholkar et al. [78]	-	-	203	50	800	46.2	1.57	6 No. 5	3.7	GS	3.2	1570
220	Sun et al. [79]	150	150	-	23.51	1103	54.1	1.5	6 No. 3	1.04	SH	0.63	201
221	Sun et al. [79]	150	150	-	23.51	1103	54.1	1.5	6 No. 3	1.04	SH	0.63	174
222	Sun et al. [79]	150	150	-	23.51	1103	54.1	1.5	6 No. 3	1.04	SH	0.63	181
223	Sun et al. [79]	150	150	-	23.51	1103	54.1	1.5	6 No. 3	1.04	SH	0.63	291
224	Sun et al. [79]	150	150	-	23.51	1103	54.1	1.5	6 No. 3	1.04	SH	0.63	290
225	Sun et al. [79]	150	150	-	23.51	1103	54.1	1.5	6 No. 3	1.04	SH	0.63	347
226	Sun et al. [79]	150	150	-	23.51	1103	54.1	1.5	6 No. 3	1.04	SH	0.63	632
227	Sun et al. [79]	150	150	-	23.51	1103	54.1	1.5	6 No. 3	1.04	SH	0.63	677
228	Sun et al. [79]	150	150	-	23.51	1103	54.1	1.5	6 No. 3	1.04	SH	0.63	602
229	Tikka et al. [80]	150	150	-	25.7	630	40	1.5	4 No. 4	2.3	CS	0.33	401
230	Tikka et al. [80]	150	150	-	25.7	630	40	1.5	4 No. 4	2.3	CS	0.33	120
231	Tikka et al. [80]	150	150	-	25.7	630	40	1.5	6 No. 4	3.4	CS	0.33	215
232	Tikka et al. [80]	150	150	-	25.7	630	40	1.5	4 No. 4	2.3	CS	0.33	382
233	Tikka et al. [80]	150	150	-	25.7	630	40	1.5	4 No. 4	2.3	CS	0.33	129
234	Tikka et al. [80]	150	150	-	25.7	630	40	1.5	6 No. 4	3.4	CS	0.33	220
235	Tikka et al. [80]	150	150	-	25.7	630	40	1.5	6 No. 4	3.4	CS	0.33	116
236	Tobbi et al. [22]	350	350	-	32.6	728	47.6	1.53	8 No. 6	1.9	GH	2	3929
237	Tobbi et al. [22]	350	350	-	32.6	728	47.6	1.53	8 No. 6	1.9	GH	2	3991
238	Tobbi et al. [22]	350	350	-	32.6	728	47.6	1.53	9 No. 6	1.9	GH	1.7	4006
239	Tobbi et al. [22]	350	350	-	32.6	752	48.2	1.56	12 No. 5	1.9	GH	3.2	3938
240	Tobbi et al. [22]	350	350	-	32.6	751	48.2	1.56	12 No. 5	1.9	GH	4.8	4067
241	Tobbi et al. [22]	350	350	-	36.4	750	48.2	1.56	8 No. 6	1.9	GH	2.55	4297
242	Tobbi et al. [22]	350	350	-	36.4	749	48.2	1.56	12 No. 5	1.9	GH	3.41	4615
243	Tobbi et al. [22]	350	350	-	36.4	748	48.2	1.56	4 No. 4 + 4 No. 5	1	GH	2.55	4212
244	Tobbi et al. [22]	350	350	-	36.4	747	48.2	1.56	8 No. 4	0.8	GH	2.55	3900
245	Tu et al. [81]	200	200	-	32.1	660	44.25	1.52	4 No. 4	1.1	GH	5.3	970.9
246	Tu et al. [81]	200	200	-	32.1	660	44.25	1.52	4 No. 4	1.1	GH	3.1	951.6
247	Tu et al. [81]	200	200	-	32.1	660	44.25	1.52	4 No. 4	1.1	GH	2	937.7
248	Tu et al. [81]	200	200	-	32.1	735	46	1.6	4 No. 3	0.8	GH	3.1	936.8
249	Tu et al. [81]	200	200	-	32.1	660	44.25	1.52	4 No. 4	1.5	GH	3.1	981.7
250	Tu et al. [81]	200	200	-	32.1	660	44.25	1.52	4 No. 4	1.1	GH	5.2	954
251	Tu et al. [81]	200	200	-	32.1	660	44.25	1.52	4 No. 4	1.1	GH	3	943.2
252	Tu et al. [81]	200	200	-	32.1	660	44.25	1.52	4 No. 4	1.1	GH	1.9	927.7
253	Xue et al. [82]	300	300	-	39	654	39	2.1	6 No. 5	1.3	SH	0.37	3091
254	Xue et al. [82]	300	300	-	39	654	39	2.1	6 No. 5	1.3	SH	0.37	2855
255	Xue et al. [82]	300	300	-	39	654	39	2.1	6 No. 5	1.3	SH	0.37	2411
256	Xue et al. [82]	300	300	-	39	654	39	2.1	6 No. 5	1.3	SH	0.37	1900
257	Xue et al. [82]	300	300	-	39	654	39	2.1	6 No. 5	1.3	SH	0.37	647
258	Xue et al. [82]	300	300	-	39	654	39	2.1	6 No. 5	1.3	SH	0.37	806
259	Xue et al. [82]	300	300	-	39	654	39	2.1	6 No. 5	1.3	SH	0.37	1702
260	Xue et al. [82]	300	300	-	40.3	654	39	2.1	6 No. 5	1.3	SH	0.37	1678
261	Xue et al. [82]	300	300	-	40.3	654	39	2.1	6 No. 5	1.3	SH	0.37	1632
262	Xue et al. [82]	300	300	-	40.3	654	39	2.1	6 No. 5	1.3	SH	0.37	1500
263	Xue et al. [82]	300	300	-	40.3	654	39	2.1	6 No. 5	1.3	SH	0.37	1300
264	Xue et al. [82]	300	300	-	40.3	654	39	2.1	4 No. 5	0.9	SH	0.37	1564
265	Xue et al. [82]	300	300	-	40.3	729	44	2.1	8 No. 6	2.6	SH	0.37	1823
266	Xue et al. [82]	300	300	-	29.1	654	39	2.1	6 No. 5	1.3	SH	0.37	1025
267	Xue et al. [82]	300	300	-	55.2	654	39	2.1	6 No. 5	1.3	SH	0.37	2191
268	Youssef and Hadi [83]	210	210	-	29.3	405.9	23.4	1.8	4 No. 4	1.15	GH	2.24	1285
269	Youssef and Hadi [83]	210	210	-	29.3	405.9	23.4	1.8	4 No. 4	1.15	GH	2.24	803
270	Youssef and Hadi [83]	210	210	-	29.3	405.9	23.4	1.8	4 No. 4	1.15	GH	2.24	615
271	Zhang and Deng [84]	350	350	-	42.5	840	45	1.87	8 No. 5	1.39	GH	1.8	5670
272	Zhang and Deng [84]	350	350	-	42.5	840	45	1.87	8 No. 5	1.39	GH	1.8	4585
273	Zhang and Deng [84]	350	350	-	42.5	840	45	1.87	8 No. 5	1.39	GH	1.8	5361
274	Zhang and Deng [84]	350	350	-	42.5	840	45	1.87	8 No. 5	1.39	GH	2.7	5205
275	Zhang and Deng [84]	350	350	-	42.5	840	45	1.87	8 No. 5	1.39	GH	2.7	5357
276	Zhang and Deng [84]	350	350	-	42.5	840	45	1.87	8 No. 5	1.39	GH	2.7	4852
277	Zhang and Deng [84]	350	350	-	42.5	840	45	1.87	12 No. 5	2.09	GH	2.49	4500
278	Zhang and Deng [84]	350	350	-	42.5	840	45	1.87	12 No. 5	2.64	GH	2.49	4972
